# Dapagliflozin Alleviates Coxsackievirus B3-induced Acute Viral Myocarditis by Regulating the Macrophage Polarization Through Stat3-related Pathways

**DOI:** 10.1007/s10753-022-01677-2

**Published:** 2022-06-08

**Authors:** Pengcheng Yan, Xiaoning Song, Joanne Tran, Runfa Zhou, Xinran Cao, Gang Zhao, Haitao Yuan

**Affiliations:** 1 Department of Cardiology, Shandong Provincial Hospital, Shandong University, Jinan, Shandong 250021 People’s Republic of China; 2 Department of Cardiology, Shandong Provincial Hospital Affiliated to Shandong First Medical University, Jinan, Shandong 250021 People’s Republic of China; 3 University of Portland, Portland, Oregon 97239 USA

**Keywords:** dapagliflozin, macrophage polarization, viral myocarditis, Stat3

## Abstract

Viral myocarditis (VMC), which is most prevalently caused by Coxsackievirus B3 (CVB3) infection, is a serious clinical condition characterized by cardiac inflammation. Dapagliflozin, a kind of sodium glucose co-transporters 2(SGLT-2) inhibitor, exhibited protective effects on plenty of inflammatory diseases, while its effect on viral myocarditis has not been studied. Recently, we found the protective effect of dapagliflozin on VMC. After CVB3 infection, dapagliflozin and STATTIC (a kind of stat3 inhibitor) were given to Balb/c male mice for 8 days, and then the severity of myocarditis was assessed. Our results indicated that dapagliflozin significantly alleviated the severity of viral myocarditis, elevated the survival rate, and ameliorated cardiac function. Besides, dapagliflozin can decrease the level of pro-inflammatory cytokines including IL-1β, IL-6, and TNF-α. Furthermore, dapagliflozin can inhibit macrophages differentiate to classically activated macrophages (M1) in cardiac tissue and activate the Stat3 signal pathway which is reported to promote polarization of the alternatively activated macrophage (M2). And STATTIC can reverse these changes caused by dapagliflozin. In conclusion, we found that dapagliflozin treatment increased anti-inflammatory macrophage polarization and reduced cardiac injury following VMC via activating Stat3 signal pathway.

## INTRODUCTION

Myocarditis can be caused by a variety of pathogens including virus, bacteria, fungi, Chlamydia, rickettsia, protozoa, and toxins. Viral myocarditis (VMC) is mainly caused by enterovirus, especially coxsackievirus group B3 (CVB3). In recent years, the incidence of VMC is increasing steadily in the whole word, although most viral myocarditis is self-limited, some acute myocarditis may develop into chronic stage, finally generating dilated cardiomyopathy (DCM) and congestive heart failure [1, 2]. The pathogenesis of VMC induced by CVB3 includes direct viral injury and indirect injury caused by immune response. After CVB3 infection, numerous of inflammatory factors are produced such as IFN-γ, IL-1β, and TNF-α, which will lead to the migration and infiltration of macrophages, and eventually lead to autoimmune injury [3, 4].

There is a remarkable gender difference in the type of cardiac macrophages in VMC. The macrophages infiltrated in the heart of female mice are mainly M2-type, which secretes anti-inflammatory factors such as IL-10. While in male mice are mainly M1-type, which secretes pro-inflammatory factors such as IL-6, IL-12, and chemokine MCP-1 [5, 6]. Thus, inhibiting macrophage polarizing to M1-type or promoting macrophage polarizing to M2-type can alleviate the myocardial inflammation in male mice [7, 8].

Dapagliflozin, a kind of sodium glucose co-transporters 2 (SGLT-2) inhibitor, targets renal glucose reabsorption. Recently, several clinical trials showed the cardiovascular protective effects of SGLT-2 inhibitors [9]. Both DAPA-HF and EMPEROR-Reduced trials showed that in patients with heart failure, SGLT2 inhibitor reduced the combined risk of cardiovascular death or hospitalization for heart failure [10, 11], and consistent with the above clinical trials, several studies indicated that the benefits of SGLT-2 inhibitors depending on its anti-inflammatory effects [12–14]. SGLT-2 inhibitors can reduce inflammatory markers such as interleukin-6 (IL-6), monocyte chemoattractant protein-1 (MCP-1), and tumor necrosis factor (TNF-α), thereby alleviating arteriosclerosis in diabetes animal models [15]. Terami *et al*. treated diabetic mice with dapagliflozin and found that macrophage infiltration in the treatment group and the expressions of inflammatory genes such as MCP-1 and transforming growth factor-β (TGF-β) all decreased [16]. Weiling Leng *et al*. found that dapagliflozin has a therapeutic effect on diabetic atherosclerosis, which may depend on macrophages [17].

The transcription factor signal transducer and activator of transcription 3 (STAT3) is recognized to have protective effects in the heart [18]. There is growing evidence indicating that STAT3 signaling pathway can regulate the phenotype of macrophages, which STAT3 is a key factor in the polarization of M2 [5, 19]. Coincidentally, Tsung-Ming Lee *et al*. reported that dapagliflozin induced M2 polarization through a RONS-dependent STAT3-mediated pathway in the infarcted rat heart [20]. Furthermore, cardiac-specific STAT3 knockout mice showed a long-term decline in cardiac function after virus infection, which is associated with myocardial fibrosis [21].

These suggested that dapagliflozin plays an important role in inflammatory disease. However, the role of dapagliflozin in CVB3 induced viral myocarditis has not been reported. In view of this, we would like to explore the role of dapagliflozin in VMC induced by CVB3 and the specific molecular biological mechanism.

## METHODS

### Animals and Virus

Six-week-old male BALB/c mice were purchased from Vital River Laboratory Animal Technology (Beijing, China). All animals were fed in the Experimental Animal Center of Shandong Provincial Hospital (Shandong, People’s Republic of China). All animal experiments and procedures were approved by the Ethics Committee of Shandong Provincial Hospital, and the experiments were conducted in accordance with the Guide for the Care and Use of Laboratory Animals, 8th edition, published by the National Institutes of Health (NIH Publication No. 85–23, revised 1996) [22]. The CVB3 (Nancy strain) was prepared by passage through HeLa cell cultures. Virus titers were determined through a 50% tissue culture infectious dose (TCID50) assay of HeLa cells and calculated by the Reed–Muench method [23].

### Experiment (*In vivo*)

Forty-eight male mice (18–20 g, 6 weeks old) were randomly assigned into 4 groups with 12 mice per group: Control, Dapa (dapagliflozin, a specific SGLT2 inhibitor, Bristol-Myers Squibb), CVB3, and the combination of CVB3 + Dapa. Mice were injected with 100 µl of phosphate-buffered saline (PBS) containing 10^3^ TCID50 of CVB3 intraperitoneally at day 0 to establish a VMC model as previously described in the literature, while control mice were injected with 100 µl of PBS. Dapagliflozin (0.1 mg/kg per day) dissolved in 60% propylene glycol or control agent (60% propylene glycol) was given daily by gavage from day 1. To further investigate whether dapagliflozin alleviates myocarditis by increasing stat3 phosphorylation, 36 male mice (18–20 g, 6 weeks old) were injected 10^3^ TCID50 of CVB3 intraperitoneally at day 0 to establish a VMC model, and then randomized to either CVB3, CVB3 + Dapa, or CVB3 + Dapa + STATTIC. STATTIC (MedChemExpress,10 mg/kg) was given every 2 days intraperitoneally according to the instructions from day 1. At day 8, all the mice underwent echocardiography and were then executed, heart tissues and serum were taken for the next analysis.

### Cell Culture and Treatment Protocol

The Raw264.7 macrophage cell line was cultured in RPMI-1640 (Life Technologies, Carlsbad, CA, USA) supplemented with 10% fetal bovine serum (FBS) (Life Technologies). 1 × 10^6^ cells/well were seeded in 6-well plate and incubated in serum free medium overnight before treatment. To examine the effect of dapagliflozin, lipopolysaccharides (LPS) (Sigma) were administered to stimulate M1 macrophage activation, dapagliflozin (10 μM) was given at the same time; after 24 h stimulation, the macrophages’ phenotype distribution and inflammatory factors were observed.

### Mouse Cardiac Echocardiography

Mouse cardiac echocardiography was operated in accordance with the manufacturers’ instructions. Mice were anesthetized by 2% isoflurane (supplied by Shandong Provincial Hospital). Then, the cardiac function parameters such as left ventricular internal dimensions in systole (LVID-s) and diastole (LVID-d), left ventricular fractional shortening (LVFS), and left ventricular ejection fraction (LVEF) were measured. The experiment was conducted in a double-blind fashion.

### Serological Index Measurement

Serum cardiac troponin I (cTNI) activities were tested by Shandong Provincial Hospital. The levels of serum IL-1β, IL-6, and TNF-α were determined by ELISA (eBioscience) following the manufacturer’s instructions. All samples were measured in triplicate.

### Histopathological Analysis

Heart samples were fixed in 10% buffered formalin solution and then embedded in paraffin. The samples were sectioned (5 μm thick) and then stained with hematoxylin and eosin (Servicebio, Wuhan,China) according to the manufacturing instructions, and the degree of inflammation was determined under 200 × magnification light microscopy. Myocardial histopathologic findings were quantified and scored for severity as follows: 0 = no inflammation, 1 = one to five distinct mononuclear inflammatory foci with 5% involvement or less of the cross-sectional area, 2 = more than five distinct mononuclear inflammatory foci or over 5% but not exceeding 20% involvement of the cross-sectional area, 3 = diffuse mononuclear inflammation involving over 20% of the area without necrosis, and 4 = diffuse inflammation with necrosis. The analysis was performed in a double-blinded manner by a trained pathologist. Besides, myocardial fibrosis was determined by Masson staining (Servicebio, Wuhan,China) according to the manufacturing instructions. Myocardial cells were stained red and collagenous fibers were stained blue. Collagen area fraction (collagen area/field area × 100%) was calculated by the Image-Pro Plus analysis system.

### Immunofluorescence

Heart samples were fixed in 10% buffered formalin solution and then embedded in paraffin. The samples were sectioned (5 μm thick). Incubate sections in 2 changes of xylene, 15 min each. Dehydrate in 2 changes of pure ethanol for 5 min, followed by dehydrating in gradient ethanol of 85% and 75% ethanol, respectively, 5 min each. Wash in distilled water. Immerse the slides in EDTA antigen retrieval buffer (pH 8.0) and maintain at a sub-boiling temperature for 8 min, standing for 8 min and then followed by another sub-boiling temperature for 7 min. Let air cool. Wash three times with PBS (pH 7.4) in a Rocker device, 5 min each. Block endogenous peroxidase: wash three times with PBS (pH 7.4) in a Rocker device, 5 min each eliminate obvious liquid, mark the objective tissue with liquid blocker pen. Immerse in 3% H_2_O_2_ and incubate at room temperature for 25 min, kept in a dark place. Then, wash again three times with PBS (pH 7.4) in a Rocker device, 5 min each. Eliminate obvious liquid, and mark the objective tissue with liquid blocker pen. Cover objective tissues with 10% donkey serum at room temperature for 30 min. Incubate slides with CD68 antibody (diluted at 1:200; Servicebio) overnight at 4 °C, wash slides three times with PBS (pH 7.4) in a Rocker device, 5 min each. Cover objective tissue with Cy3 conjugated Goat Anti-Rabbit IgG (diluted at 1:200; Servicebio), and incubate at room temperature for 50 min in dark conditions. Wash slides three times with PBS (pH 7.4) in a Rocker device, 5 min each. Incubate slides with TSA-FITC solution for 10 min in dark conditions. After that, wash slides three times with TBST in a Rocker device, 5 min each. Immerse the slides in EDTA antigen retrieval buffer (pH 8.0) and maintain at a sub-boiling temperature for 8 min, standing for 8 min and then followed by another sub-boiling temperature for 7 min, to remove the antibodies combined with tissue. Incubate slides with CD163 antibody (diluted at 1:1000; Servicebio) overnight at 4 °C, placed in a wet box containing a little water. Wash slides three times with PBS (pH 7.4) in a Rocker device, 5 min each. Then throw away the liquid slightly. Cover objective tissue with Cy3 conjugated Goat Anti-Rabbit IgG (diluted at 1:200; Servicebio), incubate at room temperature for 50 min in dark conditions. Eliminate obvious liquid, incubate slides with spontaneous fluorescence quenching reagent for 5 min, then wash slides under flowing water for 10 min. Incubate with DAPI solution at room temperature for 10 min, kept in a dark place. Wash three times with PBS (pH 7.4) in a Rocker device, 5 min each. Throw away liquid slightly, then coverslip with anti-fade mounting medium. Microscopy detection and collect images by Fluorescent Microscopy. DAPI glows blue by UV excitation wavelength 330–380 nm and emission wavelength 420 nm; FITC glows green by excitation wavelength 465–495 nm and emission wavelength 515–555 nm; CY3 glows red by excitation wavelength 510–560 nm and emission wavelength 590 nm.

### Quantitative Real-Time PCR

Total RNA samples of myocardial infiltrating macrophages and cultured RAW264.7 were extracted with TRIzol reagent (Takara, China) in accordance with the manufacturer’s instruction. First-strand complementary DNA was synthesized using 1 μg of total RNA in a 20-µL reaction buffer containing MMLV-RT and oligo (dT) primers (Takara, China). The mixture was incubated at 42 °C for 60 min, 70 °C for 15 min, and then cooled to 4 °C. To detect the expression of genes (TNF-α, iNOS, CD206, Arginase-1 and Stat3), cDNA was amplified with specific real-time PCR primers by using SYBR green real-time PCR kits (Takara, China). The following mRNA primer sequences were used:

**Table:** 

Gene	Forward primer	Reverse primer
TNF-α	5′–TGTGCTCAGAGCTTTCAACAA–3′	5′–CTTGATGGTGGTGCATGAGA–3′
IL-1β	5′-GCAACTGTTCCTGAACT–3′	5′–ATCTTTTGGGGTCCGTCAACT–3′
iNOS	5′–CGAAACGCTTCACTTCCAA–3′	5′–TGAGCCTATATGCTGTGGCT–3′
CD206	5′–ACGAGCAGGTGCAGTTTACA–3′	5′–ACATCCCATAAGCCACCTGC–3′
Stat3	5′-ACGAAAGTCAGGTTGCTGCT-3′	5′–GCTGCCGTTGTTAGACTCCT–3′
β-actin	5′-TGTTACCAACTGGGACGACA–3′	5′–CTGGGTCATCTTTTCACGGT–3′

The quantified data were analyzed using the 2^−ΔΔCt^ method [24].

### Western Blot Analysis

Fresh myocardial tissue was lysed by RIPA lysis buffer (Nanjing, China) and proteins were extracted. Proteins were separated by SDS-PAGE and transferred to PVDF membranes. The membrane was closed with 5% bovine serum albumin and then incubated with the appropriate primary antibody overnight at 4 °C, followed by washing with TBS-Tween and incubation with the appropriate secondary antibody at room temperature for 1 h. Spots were detected using an ECL system (Amersham, UK) and optical density was measured using ImageJ software. The following antibodies were used: iNOS (Cell Signaling Technology, 13120S), CD206 (Abcam, ab125028), (Abcam, ab28946), IL-6 (Cell Signaling Technology, 12912S), Stat3 (Cell Signaling Technology, 9139S), Phospho-Stat3 (Cell Signaling Technology, 9145S), GAPDH polyclonal antibody (Proteintech, No.10494–1-AP).

### Statistical Analysis

The survival rate was analyzed using the log-rank test. Comparisons between two groups were performed with an unpaired *t*-test. For comparisons of four or three groups, one-way ANOVA analysis was used, followed by post-test using Tukey multiple comparison test. Correlations were determined by Pearson’s correlation coefficients. All statistical analyses were performed using the commercially available software SPSS version 20.0, GraphPad Prism version 7. The data are presented as the mean ± SD in the figures, with P < 0.05 considered significant.

## RESULTS

### Dapagliflozin Reduced Mortality and Alleviated the Cardiac Lesion of Mice with Viral myocarditis

**Fig. 1:**
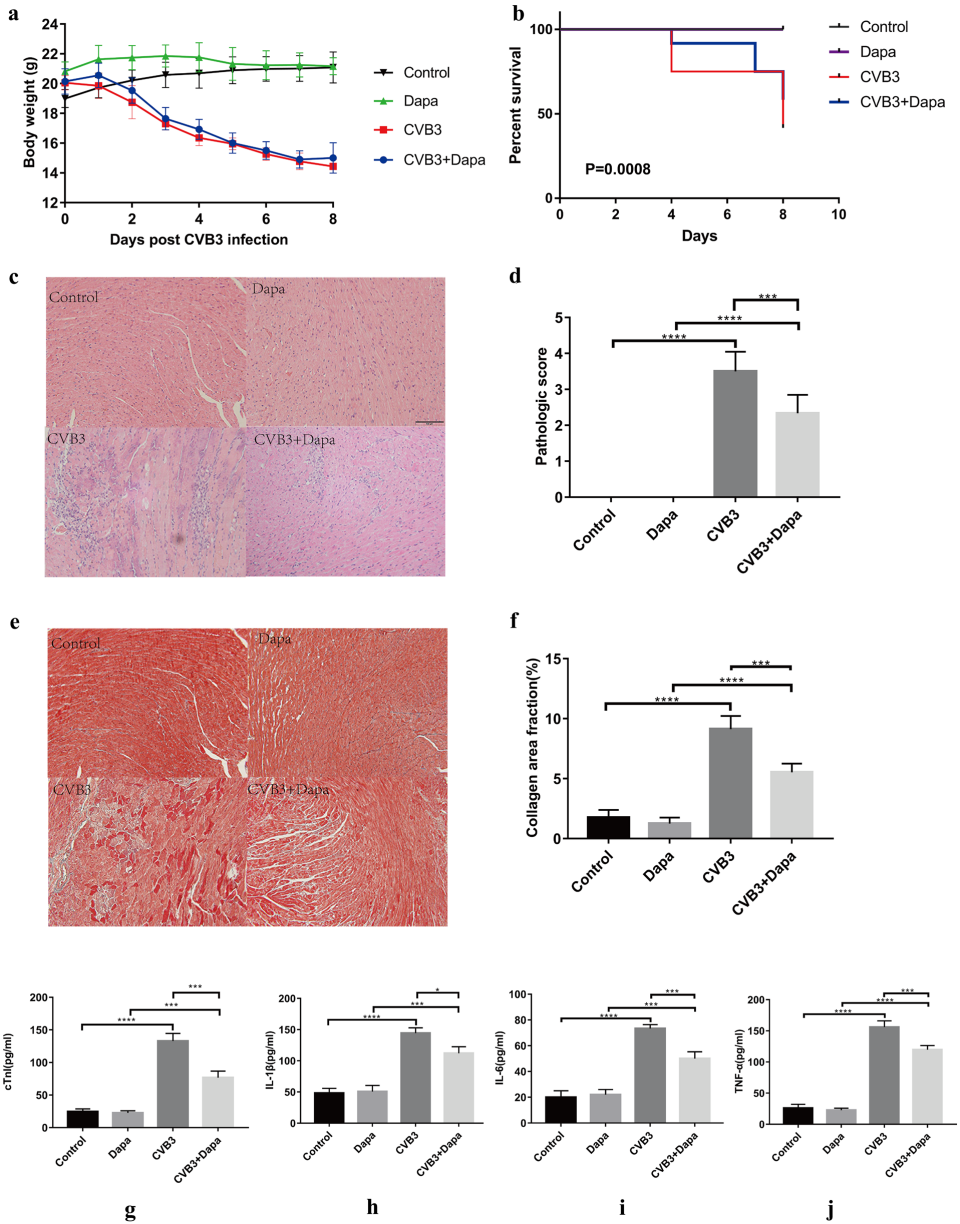
Dapagliflozin treatment ameliorates myocarditis. Viral myocarditis model was created in BALB/C mice as described in Methods. Monitor the **a** body weight change and **b** survival rate of mice from day 0 to day 8. Survival proportions at day 8 were 100% for the control group and Dapa group. **c** Hematoxylin–eosin staining to observe the inflammatory response to myocarditis. Red-stained area shows myocardial tissue, blue staining shows inflammatory cell infiltration (magnification: × 200, scale bar: 100 µm). **d** The severity of myocarditis was scored using a standard 0–4 grading scale. **e** Masson staining to observe the inflammatory response to myocarditis. Myocardial cells were stained red and collagenous fibers were stained blue (magnification: × 200, scale bar: 100 µm). **f** Collagen area fraction (collagen area/field area × 100%) was calculated by the Image-Pro Plus analysis system. **g** Serum myocardial injury markers cardiac troponin I (CTnI) were measured by ELISA. **h**, **i**, **j** Serum inflammation markers IL-1β, IL-6, and TNF-α were measured by ELISA. Data are shown as mean ± SD. (control, normal mice; Dapa, normal mice treated with dapagliflozin; CVB3, CVB3-infected mice treated with 60% propylene glycol; CVB3 + Dapa, CVB3-infected mice treated with dapagliflozin. *P < 0.05, **P < 0.01, ***P < 0.001, ****P < 0.0001).

As expected, the symptoms of viral myocarditis in the CVB3 group were evident, including a rough gray color, reduced activity and diet, and dyspnea at rest. These signs were improved to varying degrees after dapagliflozin treatment. We also monitored weight loss, the bodyweight of the CVB3 group decreased continuously from day 2 post-infection compared to the control group, and dapagliflozin has no effect on this change (Fig. 1a). No mice died in the control and Dapa groups, whereas 7 mice died in CVB3 group and 5 mice died in CVB3 + Dapa group. Dapagliflozin treatment significantly improved the survival rate of mice. (Fig. 1b). Taken together, dapagliflozin application significantly prevented the progression of CVB3-induced general presentation and improved survival.

We further assayed the potential of dapagliflozin to inhibit CVB3-mediated cardiac dysfunction by echocardiography on day 8. As Table 1 depicted, CVB3 infection led to significantly reduced cardiac contractility and impaired diastolic function, while dapagliflozin administration could reverse cardiac function decline. Thus, these data support the notion that dapagliflozin participates in CVB3-induced myocardial dysfunction *in vivo*.

**Table 1:** Dapagliflozin Alleviates CVB3 Infection Induced Reduced Cardiac Contractility and Impaired Diastolic Function

	**Control**	**Dapa**	**CVB3**	**CVB3 + Dapa**
LVID-d(Mm)	**2.66 ± 0.25**	**2.65 ± 0.31**	3.88 ± 0.21^***^	**3.36 ± 0.20**^**###**^
LVID-s(Mm)	**1.91 ± 0.37**	**1.90 ± 0.21**	2.80 ± 0.15^***^	**2.49 ± 0.06**^**###**^
LVEF (%)	**74.17 ± 6.26**	**74.88 ± 5.53**	39.76 ± 6.57^****^	**58.79 ± 6.08**^**####**^
LVFS (%)	**43.98 ± 2.85**	**44.82 ± 3.54**	24.27 ± 1.67^****^	**35.93 ± 1.87**^**####**^

Data are expressed as mean ± SD

*CVB3* coxsackievirus B3, *Dapa* dapagliflozin, *LVID-d and LVID-s* left ventricular diastolic and systolic internal diameters, *LVEF* left ventricular ejection fraction, *LVFS* left ventricular short-axis shortening rate, *Control* normal mice, *Dapa* normal mice treated with dapagliflozin, *CVB3* CVB3-infected mice treated with 60% propylene glycol, *CVB3* + *Dapa* CVB3-infected mice treated with dapagliflozin

^*^CVB3 group versus the control group, *P < 0.05; **P < 0.01; ***P < 0.001; ****P < 0.0001

^#^CVB3 + Dapa group versus the CVB3 group, #*P* < 0.05; ##*P* < 0.01; ###*P* < 0.001; ####*P* < 0.0001

Next, we examined the protective role of dapagliflozin against myocarditis-affected inflammation and cardiac remodeling *in vivo* on day 8. As shown in Fig. 1c, virus myocarditis elicited typical cardiac histopathological changes in the myocardium. Severe inflammatory lesions infiltrated by large numbers of inflammatory cells were observed, while dapagliflozin treatment significantly reduced the infiltration of inflammatory cells. Further quantitative evaluation revealed a significant reduction of mean pathological myocarditis scores in the CVB3 + Dapa group compared with the CVB3 group during the acute phase of virus myocarditis (*P* < 0.001, Fig. 1d). Consistent with these findings, the serum cTnI activities were also reduced in the mice treated with dapagliflozin (*P* < 0.001, Fig. 1g). For evaluation of cardiac remodeling, heart sections were stained with Masson trichrome dye to visualize interstitial collagen content. As shown in Fig. 1e, CVB3 resulted in a visible fibrosis in interstitial tissue of myocardium in the CVB3 group. Quantification of fibrosis (Fig. 1f) also showed a statistical increase in collagen deposition of virus mice. Contrary to this, in dapagliflozin treatment mice, the collagen area fraction was significantly lower than that in the CVB3 group (*P* < 0.001, Fig. 1e, f). Taken together, our data indicate that dapagliflozin protected mice against the pathological changes of viral myocarditis.

### Dapagliflozin Decreased the Percentage of M1 and Increased the Percentage of M2 in Myocardium at Day 8 After CVB3 Infection

**Fig. 2:**
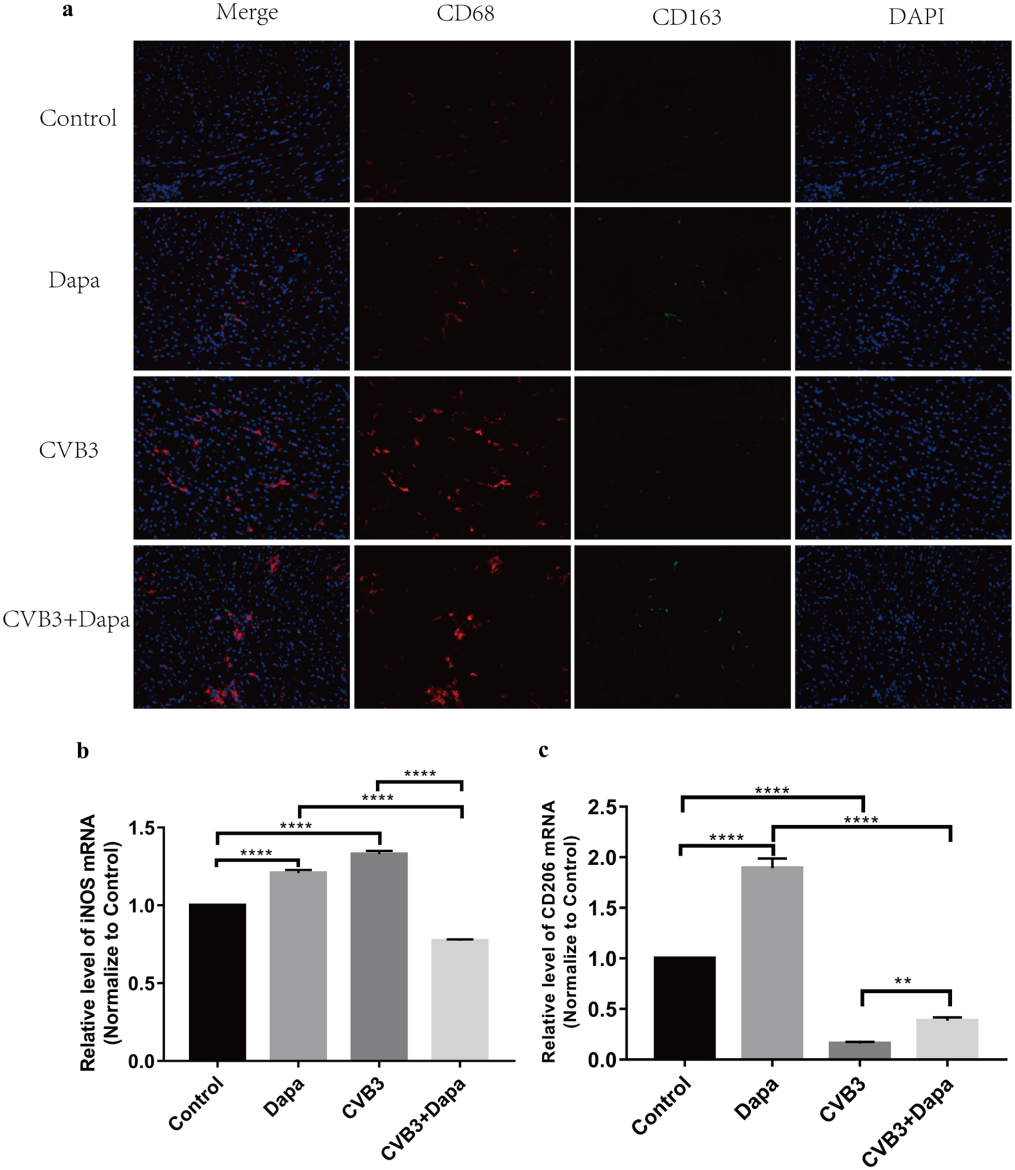
Dapagliflozin decreased the percentage of M1 and increased the percentage of M2 in myocardium at day8 after CVB3 infection. **a** Immunofluorescent double staining of myocardial tissue with antibodies to CD68 and CD163. Red indicates CD68 (one marker for M1), green CD163 (one marker for M2), and blue DAPI-stained cellular nuclei. **b**, **c** Quantitative reverse transcription polymerase chain reaction (qRT-PCR) was used to detect iNOS and CD206 mRNA levels. Data were expressed as mean ± SD (control, normal mice; Dapa, normal mice treated with dapagliflozin; CVB3, CVB3-infected mice treated with 60% propylene glycol; CVB3 + Dapa, CVB3-infected mice treated with dapagliflozin. *P < 0.05, **P < 0.01, ***P < 0.001, ****P < 0.0001).

The phenotype of macrophages is directly regulated by the composition of the microenvironment, including cytokine profiles. We therefore examined the levels of inflammatory or anti-inflammatory cytokines in each group. Plasma samples were collected at day 8 and analyzed by ELISA. The levels of IL-1β, IL-6, and TNF-α, cytokines related to macrophage, were higher in the virus group than in the control group, but were lower in dapagliflozin-treated mice (Fig. 1h, i, j). To identify the subtype of infiltrated macrophages in myocardium, the marker for M1 (CD68+) and M2 (CD163+) was examined. Immunofluorescence showed that there were a large number of CD68 + macrophages infiltrated in the CVB3 groups, and CD163 + macrophages were more frequent in the CVB3 + Dapa group (Fig. 2a). Besides, the mRNA expression of iNOS (a marker of M1 macrophages) were significantly upregulated in the myocardial tissue of mice in the CVB3 group compared with the control group (Fig. 2b), which was significantly downregulated in the dapagliflozin-treated group compared with the CVB3 group (Fig. 2b). In addition, we found that the mRNA expression of CD206 (a marker of M2 macrophages) was significantly decreased in VMC mice, and dapagliflozin treatment alleviated this change (Fig. 2c). Besides, the level of iNOS and IL-6 were significantly upregulated in the myocardial tissue of mice in the CVB3 group compared with the control group, while it was significantly downregulated in the CVB3 + Dapa group compared with the CVB3 group (Fig. 4c, d). These results suggest that dapagliflozin decreased the percentages of M1 and increased the percentages of M2 in VMC mice.

### Dapagliflozin Alleviates Myocarditis Through Activating Stat3 Signal Pathway

Previous studies have demonstrated that Stat3 is important in regulating the phenotype of macrophages, here we investigate the effect of dapagliflozin on Stat3 in VMC. We found that pstat3/stat3 was significantly upregulated in the myocardial tissue of mice in the CVB3 group compared with the control group, and it was significantly upregulated in the CVB3 + Dapagroup compared with the CVB3 group (Fig. 4b).

**Fig. 3:**
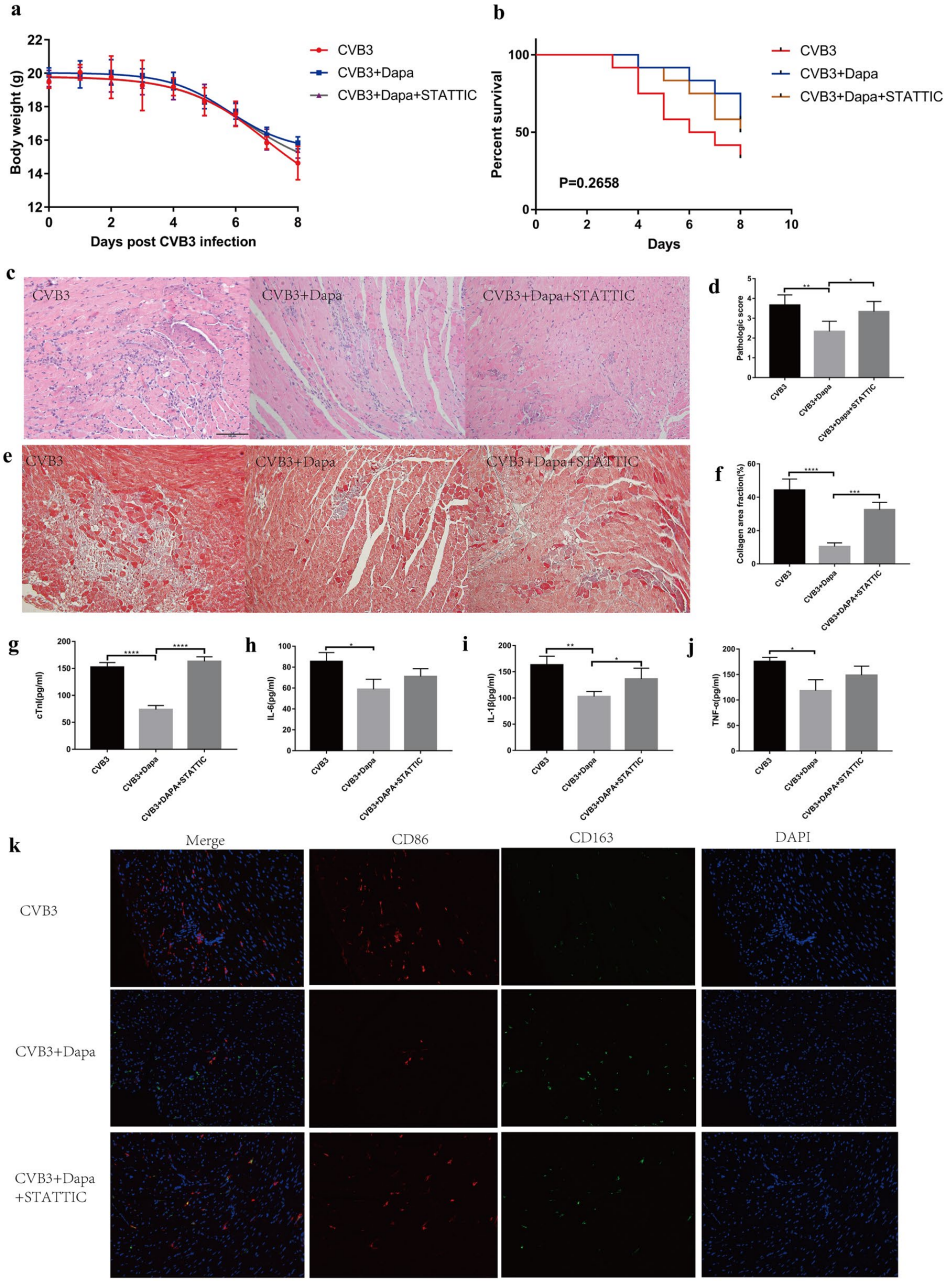
STATTIC eliminates the therapeutic effect of dapagliflozin in VMC. **a** Body weight change and **b** survival rate of mice from day 0 to day 8. **c** Hematoxylin–eosin staining to observe the inflammatory response to myocarditis. Red-stained area shows myocardial tissue, blue staining shows inflammatory cell infiltration (magnification: × 200, scale bar: 100 µm). **d** The severity of myocarditis was scored using standard 0–4 grading scale. **e** Masson staining to observe the inflammatory response to myocarditis. Myocardial cells were stained red and collagenous fibers were stained blue (magnification: × 200, scale bar: 100 µm). **f** Collagen area fraction (collagen area/field area × 100%) was calculated by the Image-Pro Plus analysis system. **g** Serum myocardial injury markers cardiac troponin I (CTnI) were measured by ELISA. **h**, **i**, **j** Serum inflammation markers IL-1β, IL-6, and TNF-α were measured by ELISA. **k** Immunofluorescent double staining of myocardial tissue with antibodies to CD68 and CD163. Red indicates CD68 (one marker for M1), green CD163 (one marker for M2), and blue DAPI-stained cellular nuclei. Data were shown as mean ± SD (CVB3, CVB3-infected mice treated with 60% propylene glycol; CVB3 + Dapa, CVB3-infected mice treated with dapagliflozin; CVB3 + Dapa + STATTIC, CVB3-infected mice treated with dapagliflozin and STATTIC, *P < 0.05, **P < 0.01, ***P < 0.001, ****P < 0.0001).

**Fig. 4:**
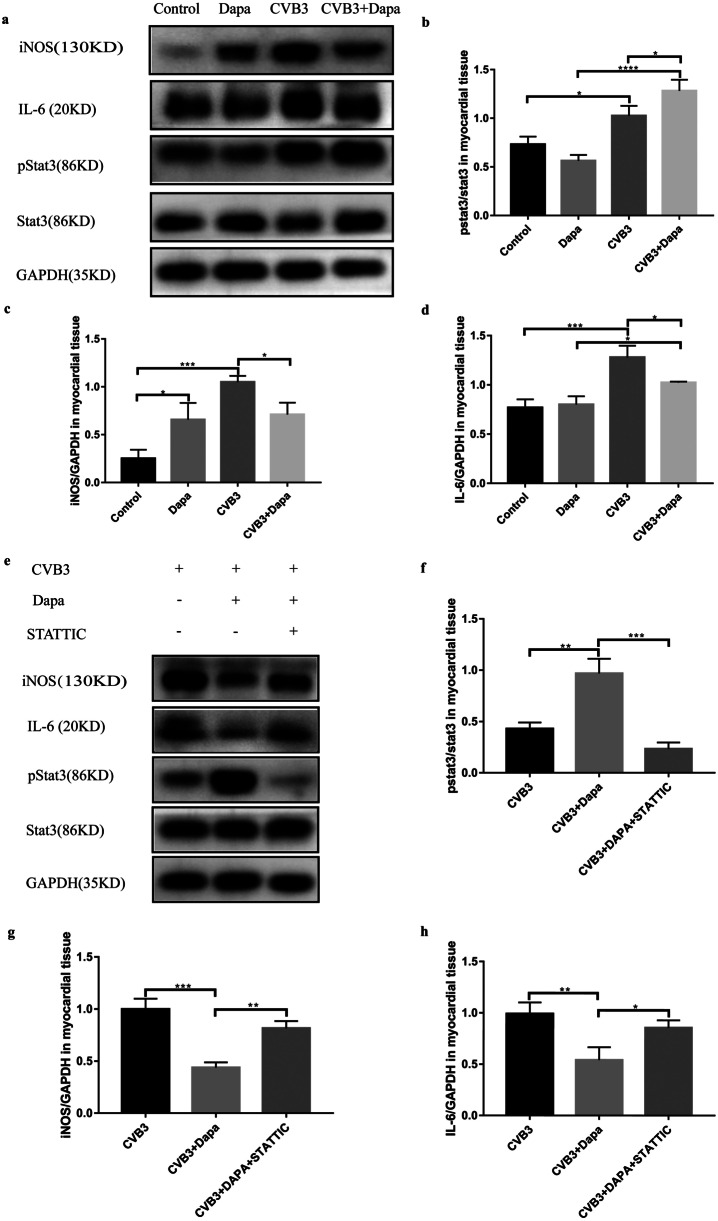
Dapagliflozin alleviates myocarditis through activating stat3 signal pathway. **a** Western blot detection of iNOS, IL-6, pstat3, stat3, and GAPDH expression. Statistics of **b** pstat3/stat3, **c** iNOS, **d** IL-6. **e** Western blot detection of iNOS, IL-6, pstat3, stat3, and GAPDH expression. Statistics of **f** pstat3/stat3, **g** iNOS, **h** IL-6. Data were expressed as mean ± SD. All the experiments were repeated at least three times (control, normal mice; Dapa, normal mice treated with dapagliflozin; CVB3, CVB3-infected mice treated with 60% propylene glycol; CVB3 + Dapa, CVB3-infected mice treated with dapagliflozin. CVB3 + Dapa + STATTIC, CVB3-infected mice treated with dapagliflozin and STATTIC. *P < 0.05, **P < 0.01, ***P < 0.001, ****P < 0.0001).

To further investigate whether dapagliflozin alleviates myocarditis by increasing stat3 phosphorylation, we injected STATTIC into the abdominal cavity of mice with viral myocarditis. Compared with the CVB3 + Dapa group, the body weight and survival rate of the CVB3 + Dapa + STATTIC group were a little bit decreased though without significance (Fig. 3a, b). What’s more, the LVFS and LVEF of STATTIC-treated myocarditis mice were significantly reduced, indicating that ventricular function worsened after STATTIC treatment (Table 2). Besides, HE and Masson stain showed more severe inflammation and fibrosis after STATTIC-treated (Fig. 3c, d, e, f,). Consistent with this, the serum cTnI activities and IL-1β were also increased in the mice treated with STATTIC (Fig. 3g, i). However, the level of IL-6 and TNF-α did not show a similar change (Fig. 3h, j). In addition, STATTIC increased the number of CD68 + macrophages and reduced CD163 + macrophages in myocardium (3 k). Furthermore, we found that STATTIC can eliminate the upregulation of p-STAT3 induced by dapagliflozin (Fig. 4e, f), while it can also eliminate the downregulation of iNOS and IL-6 induced by dapagliflozin (Fig. 4e, g, h). These data illustrated that downregulation of activated STAT3 can significantly aggravate the severity of CVB3-induced myocarditis, eliminating the therapeutic effect of dapagliflozin, which indicates that dapagliflozin alleviate myocarditis through activating stat3 signal pathway.

**Table 2:** Stat3 is involved in Dapagliflozin allviated Reduced Cardiac Contractility and Impaired Diastolic Function Caused by CVB3 Infection

	**CVB3**	**CVB3 + Dapa**	**CVB3 + Dapa + STATTIC**
LVID-d(Mm)	**4.16 ± 0.32**	3.51 ± 0.25^**^	**3.87 ± 0.13**^**#**^
LVID-s(Mm)	**3.28 ± 0.27**	2.51 ± 0.04^****^	**2.87 ± 0.15**^**###**^
LVEF(%)	**32.91 ± 3.75**	51.59 ± 3.64^****^	**38.16 ± 4.27**^**###**^
LVFS(%)	**22.45 ± 1.32**	33.96 ± 2.25^****^	**25.20 ± 1.88**^**####**^

Data are expressed as mean ± SD

*CVB3* coxsackievirus B3, *Dapa* dapagliflozin, *LVID-d and LVID-s* left ventricular diastolic and systolic internal diameters, *LVEF* left ventricular ejection fraction, *LVFS* left ventricular short-axis shortening rate, *CVB3* CVB3-infected mice treated with 60% propylene glycol, *CVB3* + *Dapa* CVB3-infected mice treated with dapagliflozin, *CVB3* + *Dapa* + *STATTIC* CVB3-infected mice treated with dapagliflozin and STATTIC

^*^CVB3 + Dapa group versus the CVB3 group, *P < 0.05, **P < 0.01, ***P < 0.001, ****P < 0.0001

^#^CVB3 + Dapa + STATTIC group versus the CVB3 + Dapa group, #*P* < 0.05, ##*P* < 0.01, ###*P* < 0.001, ####*P* < 0.0001

## DISCUSSION

Viral myocarditis is considered as an inflammatory disease and immunomodulatory therapy has drawn intensive attention. The existing literature suggests that macrophages play a crucial role in CVB3-induced myocarditis [25]. In this study, we focused on the therapeutic potential of dapagliflozin for VMC. Results showed that dapagliflozin treatment inhibited macrophage polarizing to M1 type, thereby alleviating the severity of VMC. In addition, dapagliflozin inhibited inflammatory cell infiltration and pro-inflammatory cytokine production in VMC mice infected with CVB3. And STATTIC can eliminate the therapeutic effect of dapagliflozin. These results indicate that dapagliflozin has the potential to be an effective drug for the treatment of VMC, which was depending on the activation of stat3 signal pathway.

The pathogenic mechanism of the VMC includes two sequential processes. In the early stage, the virus enters the cardiac muscle cells by receptor-mediated endocytosis. During late-stage infection, large numbers of immune cells accumulate in the infected heart tissue and strongly augment the expression of pro-inflammatory cytokines, resulting in massive inflammation and aggravated heart injury [26]. Therefore, immunosuppression is the focus of treatment research at present and many drugs have been reported have a protective effect on VMC through anti-inflammatory effects [27]. The existing literature indicates that macrophages play a crucial role in CVB3-induced myocarditis, in particular, differential-phenotype macrophages may generate an opposite inflammatory response. Consistent with this, some drugs work by promoting the polarization of macrophages towards M2 [28].

Dapagliflozin have been reported to have an anti-inflammatory function and can influence the phenotype of macrophage [17]. It has been reported that SGLT2 inhibition attenuates inflammation, including levels of CRP, IL-6, and TNF-α, and the progression of diabetic nephropathy [29–31]. A recent study suggested that empagliflozin attenuates Nlrp3 inflammasome activation in the kidney and liver of male C57BL/6 mice fed high-fat-high-sugar diet [32]. In this study, dapagliflozin treatment improved the survival rate and left ventricular function, as well as decreased the level of serum cTnI and myocardial inflammation in VMC. We observed reduced expression of iNOS (one marker of M1), as well as elevated expression of CD206 (one marker of M2), suggesting that dapagliflozin may protect VMC by inhibiting macrophage polarizing to M1.

The transcription factor signal transducer and activator of transcription 3 is an important mediator of the inflammatory process, known as a transcription activator of IL-6. Diana Lindner *et al*. found that the cardiac function in STAT3-KO mice was significantly decreased in contrast to the infected WT mice, revealing a protective function of STAT3 expressed in cardiomyocytes after CVB3-induced myocarditis. Besides, in other cardiac damages such as myocardial infarction or doxorubicin-induced cardiomyopathy, STAT3 in cardiomyocytes prevents uncontrolled fibrosis and clinical progression to DCM. Therefore, STAT3 seems to be a crucial factor for the resolution of viral myocarditis. We found that dapagliflozin treatment increased anti-inflammatory macrophage polarization and reduced cardiac injury following VMC via activating Stat3 signal pathway. However, this finding still requires further exploration due to the incompletely validated correlation between dapagliflozin and M1/M2 phenotypes seen in the current study.

Nevertheless, we still could not exclude the function of other type cells which may be involved in the viral myocarditis. RT-PCR confirmed that SGLT2 and SGLT1 are not expressed in RAW264.7 and the hearts of the Balb/c mice (data not shown), which means the anti-inflammatory, anti-fibrosis effects are likely SGLT2-independent. And this dose of dapagliflozin has been shown that blood glucose levels would not be different from those in the control group, thus enabling us to assess direct drug effects independently from blood glucose control. Although some experts tried to link the favorable effects on clinical outcomes solely to the diuretic effects of the drugs, others expressed doubts and suggested that other mechanisms may exist.

In summary, we showed for the first time that dapagliflozin can alleviate CVB3-induced myocardial inflammation, and this therapeutic effect was depended on the activation of stat3 signal pathway. Therefore, our findings suggest a therapeutic potential of dapagliflozin in the treatment of viral myocarditis.

## CONCLUSION

In the mouse model of CVB3-induced viral myocarditis, dapagliflozin inhibits the production and release of inflammatory cytokines IL-1β, IL-6, and TNF-α, inhibiting macrophage polarization toward M1-type, reducing inflammatory infiltration, and significantly improving the survival rate of mice, these effects were depended on the activation of Stat3 signal pathway.
